# Serum oxalate concentration is associated with coronary artery calcification and cardiovascular events in Japanese dialysis patients

**DOI:** 10.1038/s41598-023-45903-9

**Published:** 2023-10-29

**Authors:** Yoko Nishizawa, Satoshi Miyata, Mai Tosaka, Eriko Hirasawa, Yumi Hosoda, Ai Horimoto, Kiyotsugu Omae, Kyoko Ito, Nobuo Nagano, Junichi Hoshino, Tetsuya Ogawa

**Affiliations:** 1 Department of Medicine, Tokyo Women’s Medical University Adachi Medical Center, 4-33-1, Kohoku, Adachi, Tokyo, 123-8558 Japan; 2 Teikyo University Graduate School of Public Health, Itabashi, Tokyo, Japan; 3 Kidney Disease and Dialysis Center, Hidaka Hospital, Hidaka-kai, Takasaki, Gunma Japan; 4 Department of Nephrology, Tokyo Women’s Medical University, Shinjuku, Tokyo, Japan

**Keywords:** Cardiology, Nephrology

## Abstract

Coronary artery calcification (CAC) is associated with cardiovascular disease (CVD). CAC might contain calcium oxalate, and a high serum oxalate (S_Ox_) concentration is associated with cardiovascular mortality in dialysis patients. We assessed the associations between S_Ox_ and CAC or CVD events in Japanese hemodialysis patients. This cross-sectional and retrospective cohort study was done in 2011. Seventy-seven hemodialysis patients’ Agatston CAC score was measured, and serum samples were collected. S_Ox_ concentrations were measured in 2021 by using frozen samples. Also, new-onset CVD events in 2011–2021 were retrospectively recorded. The association between S_Ox_ concentration and CAC score ≥ 1000, and new-onset CVD events were examined. Median S_Ox_ concentration and CAC score were 266.9 (229.5–318.5) µmol/L and 912.5 (123.7–2944), respectively. CAC score ≥ 1000 was associated with S_Ox_ [adjusted odds ratio (OR) 1.01, 95% confidence interval (CI), 1.00–1.02]. The number of new-onset CVD events was significantly higher in patients with S_Ox_ ≥ median value [hazard ratio (HR) 2.71, 95% CI 1.26–6.16]. By Cox proportional hazard models, new-onset CVD events was associated with S_Ox_ ≥ median value (adjusted HR 2.10, 95% CI 0.90–4.91). S_Ox_ was associated with CAC score ≥ 1000 and new-onset CVD events in Japanese hemodialysis patients.

## Introduction

Cardiovascular disease (CVD) is a major complication of end-stage renal disease (ESRD)^1^. Vascular calcification, especially coronary artery calcification (CAC), is associated with CVD in ESRD patients^2,3^. Especially, “very high” CAC individuals, defined as Agatston CAC score^4^ ≥ 1000, are increasingly recognized as higher risk for CVD events and mortality^5–7^. A large body of evidence suggests that dysregulation of calcium and phosphate homeostasis, which are factors related to mineral and bone disorder in chronic kidney disease patients, has direct effects on vascular smooth muscle cells and promotes vascular calcification^8^. Indeed, vascular calcification in ESRD patients is formed from hydroxyapatite and calcium phosphate, both of which contain phosphate^9,10^. Phosphate binders and other treatments are available for the control of mineral and bone disorders^11^; however, even after these treatments, the prevalence of vascular calcification is still higher in ESRD patients than in the population with normal kidney function.

Studies in non-ESRD patients^12,13^ and ESRD patients^14^ indicate that CAC contains calcium oxalate crystals. Oxalate is an organic acid abundant in plants such as spinach, where it acts as a controller of calcium concentration^15^. However, in humans, it is a waste product^16^. Urine is the major excretion pathway of oxalate^17^, meaning that patients with decreased kidney function also show elevated serum oxalate (S_Ox_) concentrations, which can be 20–100 times normal in ESRD patients^18^. S_Ox_ is a small molecule, and its serum concentration can be reduced by approximately 90% per hemodialysis session, although it can easily rebound to its pre-dialysis level; for example, at only 2 h after hemodialysis treatment, S_Ox_ can already be back at its mid-dialysis level^19^. Excess S_Ox_ combines with calcium to form calcium oxalate crystals that are deposited in various tissues; for example, myocardium, renal tubules, and interstitium^20,21^. A recent study has reported a relationship between high S_Ox_ concentration and CVD mortality, especially in dialysis patients with high S_Ox_ concentrations^22^. This suggests that controlling S_Ox_ could be a novel strategy to reduce the risk of CVD in ESRD patients. However, the mechanism of how oxalate affects CVD is currently unknown, limiting the rational development of an effective therapeutic approach.

Here, we conducted analyses to examine the association between S_Ox_ and CAC and the association between S_Ox_ and CVD events in Japanese hemodialysis patients. First, we conducted a cross-sectional analysis to understand more about the relationship between high S_Ox_ concentration and CAC ≥ 1000^5–7^. Then, we conducted a retrospective cohort study to understand more about the relationship between S_Ox_ and CVD events.

## Results

### Baseline patient characteristics

Of the 77 patients that underwent the voluntary atherosclerosis checkup, 17 had normal S_Ox_ concentrations and were excluded from the study. There were no missing values except for ankle-brachial index of two cases. Of the remaining 60 patients, 41 (68.3%) were male, mean age was 63.1 ± 11.9 years, and median dialysis duration was 87.6 (44.1–152.2) months. Twenty-one patients (35%) had diabetes mellitus and 10 (16.7%) had CVD. Median S_Ox_, Agatston CAC score, and major artery calcification volume were 266.9 (229.5–318.5) µmol/L, 912.5 (123.7–2943.8), and 7.0 (3.0–18.4) cm^3^, respectively; these data were right skewed. When the subjects were divided into two groups according to CAC score ≥ 1000, baseline CVD, serum phosphate, low-density lipoprotein (LDL) cholesterol, and major artery calcification volume were significantly higher in the CAC ≥ 1000 group. Table 1 provides the baseline patient characteristics.

**Table 1:** Baseline patient characteristics.

	All (n = 60)	CAC < 1,000 (n = 33)	CAC ≥ 1,000 (n = 27)	*P*
Age (years)	63.1 ± 11.9	61.8 ± 13.1	64.7 ± 10.4	0.335*
Male	41 (68.3%)	19 (57.6%)	22 (81.5%)	0.057^†^
Hemodialysis duration (months)	87.6 (44.1, 152.2)	87.4 (42.1, 138.8)	96.9 (47.5, 165.2)	0.637^‡^
Cardiovascular disease	10 (16.7%)	0 (0%)	10 (37%)	< 0.001^†^
Diabetes mellitus	21 (35%)	12 (36.4%)	9 (33.3%)	1.000^†^
Body mass index (kg/m^2^)	21.5 (20.6, 23.2)	21.4 (20.4, 22.9)	21.5 (20.9, 23.5)	0.440^‡^
Ankle-brachial index	1.2 (1.1, 1.2)	1.2 (1.1, 1.2)	1.2 (1.1, 1.2)	0.789^‡^
Albumin-adjusted calcium (mg/dL)	9.0 ± 0.5	9.0 ± 0.5	9.0 ± 0.4	0.940*
Serum phosphate (mg/dL)	5.2 (4.6, 5.7)	4.9 (4.5, 5.2)	5.5 (4.7, 6.1)	0.020^‡^
Intact parathyroid hormone (pg/mL)	176.2 (152.1, 245.4)	171.8 (152.2, 211.7)	188.4 (156.3, 255.8)	0.352^‡^
Alkaline phosphatase (U/mL)	259.1 (205.5, 318.8)	264.3 (212.5, 346.0)	251.2 (197.4, 282.7)	0.178^‡^
Magnesium (mg/dL)	2.6 ± 0.3	2.6 ± 0.3	2.5 ± 0.3	0.174*
Triglyceride (mg/dL)	107.3 (70.6, 148.9)	94.3 (58.5, 144.8)	111.3 (96.7, 154.4)	0.099^‡^
LDL cholesterol (mg/dL)	86.2 ± 21.6	81.0 ± 21.0	92.7 ± 21.5	0.038*
HDL cholesterol (mg/dL)	40.7 (32.8, 52.6)	44.2 (34.9, 54.6)	36.8 (29.7, 51.0)	0.115^‡^
Uric acid (mg/dL)	7.1 ± 0.9	7.1 ± 0.8	7.2 ± 1.0	0.548*
Beta-2 microglobulin (µg/L)	27.1 (23.0, 29.2)	27.3 (21.2, 29.2)	27.0 (23.3, 29.1)	0.876^‡^
Blood urea nitrogen (mg/dL)	60.1 ± 10.2	59.0 ± 8.6	61.4 ± 11.9	0.397*
Hemoglobin (g/dL)	10.7 (10.3, 11.0)	10.9 (10.5, 11.1)	10.4 (10.0, 10.9)	0.023^‡^
Serum albumin (g/dL)	3.7 (3.6, 4.0)	3.7 (3.6, 4.0)	3.7 (3.6, 4.0)	0.994^‡^
Calcium carbonate intake	49 (81.7%)	27 (81.8%)	22 (81.5%)	1.000^†^
Vitamin D medication	26 (43.3%)	11 (33.3%)	15 (55.6%)	0.117^†^
Statin intake	10 (16.7%)	5 (15.2%)	5 (18.5%)	0.742^†^
S_Ox_ (μg/L)	266.9 (229.5, 318.5)	256.1 (228.2, 290.1)	280.4 (251.3, 355.5)	0.081^‡^
Major artery calcification volume (cm^3^)	7.0 (3.0, 18.4)	3.6 (1.6, 8.6)	12.2 (6.1, 31.3)	< 0.001^‡^

Continuous variables are reported as mean ± standard deviation for normally distributed data or median (inter-quartile range) for non-normally distributed data. Discrete variables are expressed as numeral (percentage). The subjects were divided into two groups according to CAC score, and statistical significance was tested by using Welch’s *t*-test for normally distributed data, the Wilcoxon rank sum test for non-normally distributed data, and Fisher’s exact test for discrete variables. A two-tailed *P* value of < 0.05 was considered to indicate statistical significance.

*CAC* coronary artery calcification, *HDL* high-density lipoprotein, *LDL* low-density lipoprotein, *S*_*Ox*_ serum oxalate concentration.

*Welch’s *t*-test; ^†^Fisher’s exact test; ^‡^the Wilcoxon rank sum test.

### Cross-sectional analysis of the association between S_Ox_ and vascular calcification

To examine the association between S_Ox_ and vascular calcification, we performed a logistic regression analysis. The patients were stratified into those with CAC score < 1000 and those with CAC score ≥ 1000. All of the patients with CVD (n = 10) were in the CAC score ≥ 1000 group; those patients were excluded from this analysis, leaving 50 patients in the analysis group.

**Figure 1:**
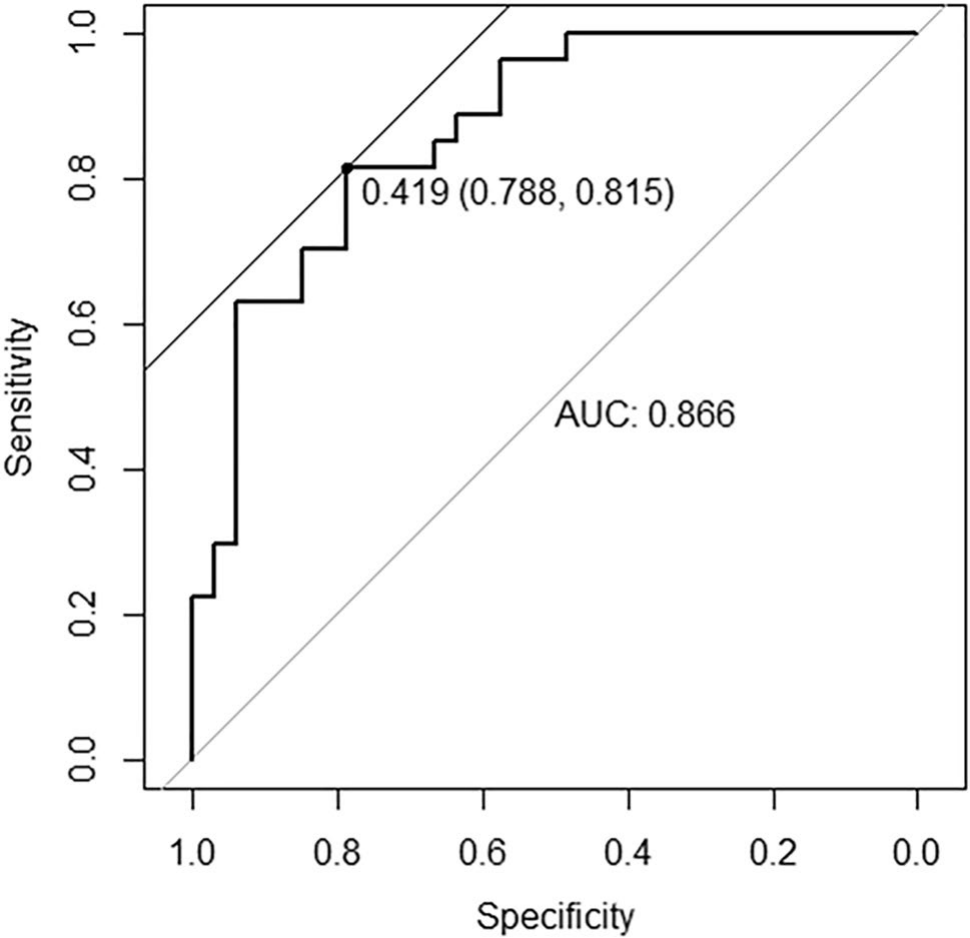
Receiving operating curve for predicting Agatston coronary artery calcification score ≥ 1000. In a risk prediction model for predicting CAC score ≥ 1000, we used five factors obtained from an earlier variable selection (i.e., S_Ox_, male, serum phosphate, intact parathyroid hormone, and LDL cholesterol). The area under the receiver operating characteristic curve was 0.87 (95%CI 0.76–0.98), and sensitivity and specificity were 81.8% and 76.5%, respectively. *AUC* area under the curve, *CAC* coronary artery calcification, *CI* confidence interval, *LDL* low-density lipoprotein, *S*_*Ox*_ serum oxalate concentration.

In a univariate analysis examining associations with CAC score ≥ 1000, variables with* P* value < 0.2 were S_Ox_ concentration, male, body mass index (BMI), serum phosphate, intact parathyroid hormone, triglyceride, LDL cholesterol, high-density lipoprotein (HDL) cholesterol, uric acid, and blood urea nitrogen (Table 2). In a multivariate analysis using these selected variables, S_Ox_ [odds ratio (OR) 1.01, 95% confidence interval (CI) 1.00–1.02, *P* = 0.168], male (OR 6.90, 95% CI 1.10–77.5, *P* = 0.065), serum phosphate (OR 2.82, 95% CI 1.17–8.94, *P* = 0.033), intact parathyroid hormone (OR 1.01, 95% CI 1.00–1.01, *P* = 0.058), and LDL cholesterol (OR 1.04, 95% CI 1.00–1.10, *P* = 0.055) were selected as factors associated with CAC score ≥ 1000 (Table 2). In a risk prediction model predicting CAC score ≥ 1000, we used the five selected factors (i.e., S_Ox_, male, serum phosphate, intact parathyroid hormone, and LDL cholesterol); the area under the receiver operating characteristic (ROC) curve was 0.87 (95% CI 0.76–0.98), and sensitivity and specificity were 81.8% and 76.5%, respectively (Fig. 1).

**Table 2:** Association between S_Ox_ and coronary artery calcification score ≥ 1000 by logistic regression analysis.

	Univariable analysis			Multivariable analysis			Variable selection (AIC)		
	Odds ratio	95% CI	*P*	Odds ratio	95% CI	*P*	Odds ratio	95% CI	*P*
S_Ox_ (µmol/L)	1.01	1.00–1.02	0.038	1.01	1.00–1.03	0.099	1.01	1.00–1.02	0.168
Age (years)	1.00	0.95–1.05	0.854						
Male	3.44	0.91–17.02	0.089	24.29	1.14–1672.3	0.076	6.90	1.10–77.5	0.065
HD duration (months)	1.00	1.00–1.01	0.235						
Diabetes mellitus	0.95	0.27–3.20	0.941						
BMI (kg/m^2^)	1.12	0.95–1.36	0.196	1.38	0.94–2.25	0.128			
Ankle-brachial index	0.80	0.01–92.26	0.923						
aCa (mg/dL)	1.36	0.43–4.63	0.604						
Phosphate (mg/dL)	3.40	1.48–10.04	0.011	2.95	0.84–14.0	0.127	2.82	1.17–8.94	0.033
iPTH (pg/dL)	1.01	1.00–1.01	0.054	1.01	1.00–1.03	0.025	1.01	1.00–1.01	0.058
ALP (U/mL)	1.00	0.99–1.00	0.773						
Magnesium (mg/dL)	0.46	0.05–3.62	0.467						
Triglyceride (mg/dL)	1.01	1.00–1.02	0.105	0.98	0.96–1.00	0.116			
LDL-C (mg/dL)	1.03	1.00–1.06	0.088	1.07	1.01–1.14	0.031	1.04	1.00–1.10	0.055
HDL-C (mg/dL)	0.96	0.92–1.01	0.126	0.95	0.83–1.04	0.337			
Uric acid (mg/dL)	1.60	0.79–3.39	0.198	2.85	0.55–20.2	0.239			
β2MG (µg/L)	1.08	0.95–1.27	0.277						
BUN (mg/dL)	1.04	0.98–1.11	0.190	0.92	0.79–1.04	0.211			
Hemoglobin (g/dL)	0.77	0.32–1.62	0.519						
Serum albumin (g/dL)	1.11	0.12–9.93	0.927						
CaCO_3_ intake	3.56	0.54–70.37	0.260						
Vitamin D medication	1.40	0.41–4.70	0.585						
Statin intake	0.75	0.10–3.94	0.744						

In the analysis, we divided subjects into two groups: those with CAC score < 1000 and those with CAC score ≥ 1000. A logistic regression analysis was performed for the association between S_Ox_ and CAC. The explanatory variable was S_Ox,_ and the covariates were factors that are reported to be associated with vascular calcification^8,25,32,33^ or CVD^29,34–36^. A univariable analysis was performed first, and factors with a two-tailed *P* value < 0.2 were used for a multivariable analysis. For variable selection, we used Akaike’s information criteria with stepwise backward elimination. A two-tailed *P* value of < 0.05 was considered to indicate statistical significance.

*aCa* albumin-adjusted calcium, *AIC* Akaike’s information criteria, *ALP* alkaline phosphatase, *β2MG* beta-2 microglobulin, *BUN* blood urea nitrogen, *BMI* body mass index, *CaCO*_*3*_ calcium carbonate, *CI* confidence interval, *HD* hemodialysis, *HDL-C* high-density lipoprotein cholesterol, *iPTH* intact parathyroid hormone, *LDL-C* low-density lipoprotein cholesterol, *S*_*Ox*_ serum oxalate concentration.

In a univariate analysis examining associations with major artery calcification volume, variables with a *P* value < 0.2 were S_Ox_, age, male, albumin-adjusted calcium, HDL cholesterol, beta-2 microglobulin, serum albumin, and calcium carbonate intake (Table 3). In a multivariate analysis using these selected variables after variable selection, S_Ox_ (β = 0.03, 95% CI 0.00–0.06, *P* = 0.084), age (β = 0.26, 95% CI 0.05–0.47, *P* = 0.018), and beta-2 microglobulin (β = 0.55, 95% CI 0.00–1.09, *P* = 0.048) were selected as factors associated with major artery calcification volume (Table 3).

**Table 3:** Association between S_Ox_ and major artery calcification volume by linear regression analysis.

	Univariable analysis			Multivariable analysis			Variable selection (AIC)		
	β	95 %CI	*P*	β	95% CI	*P*	β	95% CI	*P*
S_Ox_ (µmol/L)	0.04	0.01 to 0.07	0.022	0.02	− 0.02 to 0.06	0.264	0.03	0.00–0.06	0.084
Age (years)	0.31	0.09 to 0.54	0.007	0.29	0.03 to 0.55	0.027	0.26	0.05–0.47	0.018
Male	4.73	–1.03 to 10.50	0.105	1.98	− 4.35 to 8.31	0.531			
HD duration (months)	0.00	–0.03 to 0.03	0.972						
Diabetes mellitus	1.66	–4.17 to 7.49	0.570						
BMI (kg/m^2^)	–0.29	–1.12 to 0.53	0.478						
Ankle-brachial index	6.49	–15.71 to 28.70	0.559						
aCa (mg/dL)	3.65	–1.75 to 9.05	0.180	3.26	− 2.35 to 8.87	0.248			
Phosphate (mg/dL)	–0.26	–3.47 to 2.95	0.871						
iPTH (pg/dL)	–0.01	–0.03 to 0.02	0.545						
ALP (U/mL)	–0.01	–0.04 to 0.01	0.352						
Magnesium (mg/dL)	–2.14	–11.94 to 7.67	0.663						
Triglyceride (mg/dL)	0.00	–0.04 to 0.04	0.980						
LDL-C (mg/dL)	–0.01	–0.14 to 0.11	0.856						
HDL-C (mg/dL)	–0.16	–0.34 to 0.01	0.071	− 0.11	− 0.30 to 0.07	0.228			
Uric acid (mg/dL)	0.72	–2.66 to 4.10	0.670						
β2MG (µg/L)	0.74	0.17 to 1.30	0.013	0.42	− 0.23 to 1.06	0.200	0.55	0.00–1.09	0.048
BUN (mg/dL)	–0.16	–0.44 to 0.12	0.267						
Hemoglobin (g/dL)	–0.07	–3.55 to 3.40	0.966						
Serum albumin (g/dL)	–7.36	–17.45 to 2.73	0.149	3.95	− 7.57 to 15.47	0.492			
CaCO_3_ intake	6.53	–1.34 to 14.39	0.102	− 0.33	− 8.97 to 8.30	0.939			
Vitamin D medication	2.20	–3.62 to 8.01	0.451						
Statin intake	–5.13	–13.08 to 2.82	0.201						

A linear regression analysis was performed for the association between S_Ox_ and major artery calcification volume. The explanatory variable was S_Ox,_ and the covariates were factors that are reported to be associated with vascular calcification^8,25,32,33^ or CVD^29,34–36^. A univariable analysis was performed first, and factors with a two-tailed *P* value < 0.2 were used for a multivariable analysis. For variable selection, we used Akaike’s information criteria with stepwise backward elimination. A two-tailed *P* value of < 0.05 was considered to indicate statistical significance.

*aCa* albumin-adjusted calcium, *AIC* Akaike’s information criteria, *ALP* alkaline phosphatase, *β2MG* beta-2 microglobulin, *BUN* blood urea nitrogen, *BMI* body mass index, *CaCO*_*3*_ calcium carbonate, *CI* confidence interval, *HD* hemodialysis, *HDL-C* high-density lipoprotein cholesterol, *iPTH* intact parathyroid hormone, *LDL-C* low-density lipoprotein cholesterol, *S*_*Ox*_ serum oxalate concentration.

### Retrospective cohort analysis of the relationship between S_Ox_ and new-onset CVD events

**Figure 2:**
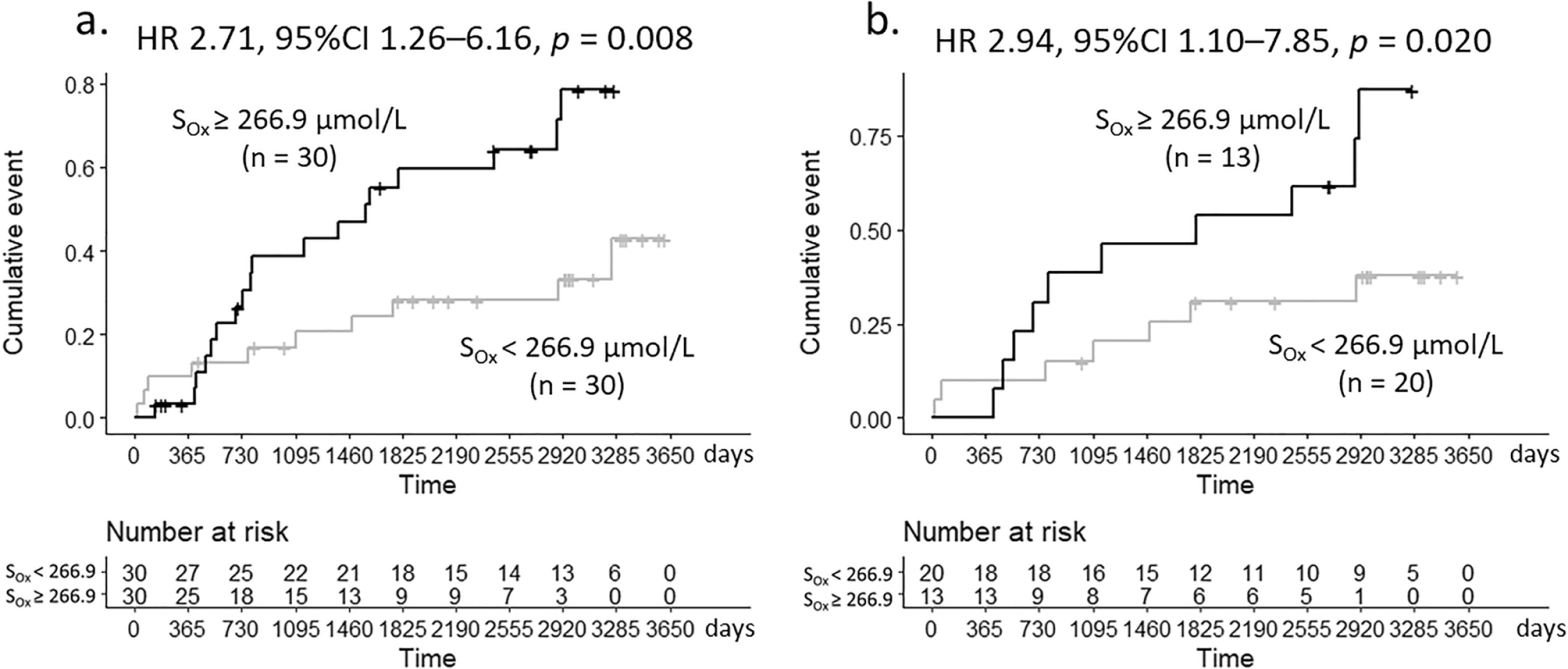
Kaplan–Meier analysis of the relationship between S_Ox_ and new-onset cardiovascular disease events. Subjects were divided into two groups by median S_Ox_ concentration (S_Ox_ < 266.9 µmol/L and S_Ox_ ≥ 266.9 µmol/L). New-onset cardiovascular events occurred in 29/60 (48.3%) patients: in 19/30 (63.3%) patients with S_Ox_ greater than or equal to the median value and in 10/30 (33.3%) patients with S_Ox_ less than the median value. The number of events was significantly higher in the patients with S_Ox_ ≥ 266.9 µmol/L than in those with S_Ox_ < 266.9 µmol/L (**a**), even after limiting the analysis only to patients with CAC score < 1000 and without history of cardiovascular disease (n = 33, **b**). *CAC* coronary artery calcification, *CI* confidence interval, * HR* hazard ratio, *S*_*Ox*_ serum oxalate.

During the 10-year observation period, seven patients (11.7%) were censored due to transfer to another dialysis center, three patients (5.0%) were censored due to kidney transplantation, and nine patients (15.0%) died before experiencing a CVD event. A total of 29 (48.3%) patients experienced new-onset CVD events. We divided the subjects into two groups using the median S_Ox_ value of 266.9 µmol/L. New-onset CVD events occurred in 19/30 (63.3%) patients with S_Ox_ greater than or equal to the median value, whereas new-onset CVD events occurred in only 10/30 (33.3%) patients with S_Ox_ lower than the median value; the risk of new-onset CVD events was greater in the patients with S_Ox_ greater than or equal to the median value than in those with S_Ox_ lower than the median value [hazard ratio (HR) 2.71, 95% CI 1.26–6.16, *P* = 0.008; Fig. 2a]. By restricting the analysis to patients with CAC score < 1000 and without history of CVD (n = 33), CVD events occurred in 10/13 (76.9%) patients with S_Ox_ greater than or equal to the median value and in 7/20 (35.0%) patients with S_Ox_ lower than the median value, respectively. The number of events was significantly higher in the patients with S_Ox_ greater than or equal to the median value than in the patients with lower than the median value (HR 2.94, 95% CI 1.10–7.85, *P* = 0.020; Fig. 2b).

We performed Cox proportional hazard analyses to examine the relationship between S_Ox_ and new-onset CVD events. In a univariate analysis, variables with a *P* value < 0.2 were S_Ox_ greater than or equal to the median value, age, male, history of CVD, alkaline phosphatase, HDL cholesterol, and hemoglobin (Table 4). In a multivariable analysis using these variables after variable selection, S_Ox_ (HR 2.10, 95% CI 0.90–4.91, *P* = 0.086), history of CVD (HR 3.84, 95% CI 1.44–10.2, *P* = 0.007), and HDL cholesterol (HR 0.97, 95% CI 0.94–1.00, *P* = 0.062) were selected as factors associated with new-onset CVD events (Table 4).

**Table 4:** Relationship between S_Ox_ and new-onset cardiovascular disease events by Cox proportional hazard analysis.

	Univariable analysis			Multivariable analysis			Variable selection (AIC)		
	HR	95%CI	*P*	HR	95%CI	*P*	HR	95%CI	*P*
S_Ox_ ≥ 266.9 (µmol/L)	2.79	1.26–6.16	0.011	1.92	0.79–4.68	0.150	2.10	0.90–4.91	0.086
Age (years)	1.02	0.99–1.06	0.159	1.00	0.96–1.04	0.951			
Male	2.07	0.87–4.89	0.099	1.34	0.53–3.39	0.531			
HD duration (months)	1.00	0.99–1.00	0.403						
History of CVD	5.88	2.28–15.15	< 0.001	3.23	1.06–9.85	0.039	3.84	1.44–10.2	0.007
Diabetes mellitus	1.41	0.65–3.07	0.390						
BMI (kg/m^2^)	1.00	0.88–1.13	0.993						
Ankle-brachial index	0.22	0.01–4.58	0.329						
aCa (mg/dL)	0.58	0.24–1.39	0.221						
Phosphate (mg/dL)	1.14	0.71–1.85	0.585						
iPTH (pg/dL)	1.00	0.99–1.00	0.324						
ALP (IU/L)	1.00	0.99–1.00	0.142	1.00	0.99–1.00	0.708			
Magnesium (mg/dL)	0.44	0.13–1.55	0.201						
Triglyceride (mg/dL)	1.00	1.00–1.01	0.306						
LDL-C (mg/dL)	1.01	0.99–1.02	0.494						
HDL-C (mg/dL)	0.96	0.93–1.00	0.030	0.97	0.94–1.00	0.091	0.97	0.94–1.00	0.062
Uric acid (mg/dL)	0.91	0.59–1.42	0.686						
β2MG (µg/L)	0.99	0.93–1.06	0.837						
BUN (mg/dL)	0.98	0.94–1.02	0.283						
Hemoglobin (g/dL)	0.64	0.35–1.16	0.139	0.82	0.45–1.48	0.508			
Serum albumin (g/dL)	0.53	0.13–2.11	0.367						
CaCO_3_ intake	0.83	0.34–2.05	0.683						
Vitamin D medication	1.33	0.63–2.81	0.451						
Statin intake	1.03	0.39–2.73	0.952						

We divided the subjects into two groups by S_Ox_ value ≥ 266.9 μmol/L and conducted a Cox proportional hazard analysis. The explanatory variable was S_Ox,_ and the covariates were factors that are reported to be associated with vascular calcification^8,25,32,33^ or CVD^29,34–36^. A univariable analysis was performed first, and factors with a two-tailed *P* value < 0.2 were used for a multivariable analysis. For variable selection, we used Akaike’s information criteria with stepwise backward elimination. A two-tailed *P* value of < 0.05 was considered to indicate statistical significance.

*aCa* albumin-adjusted calcium, *AIC* Akaike’s information criteria, *ALP* alkaline phosphatase, *β2MG* beta-2 microglobulin, *BUN* blood urea nitrogen, *BMI* body mass index, *CaCO*_*3*_ calcium carbonate, *CI* confidence interval, *CVD* cardiovascular disease, *HD* hemodialysis, *HDL-C* high-density lipoprotein cholesterol, *HR* hazard ratio, *iPTH* intact parathyroid hormone, *LDL-C* low-density lipoprotein cholesterol, *S*_*Ox*_ serum oxalate concentration.

## Discussion

In this study, higher S_Ox_ concentration was associated with vascular calcification in both the coronary artery and other major arteries, and with new-onset CVD events in Japanese dialysis patients. Several previous studies have concluded that CAC contains calcium oxalate^12–14^; however, no studies have examined S_Ox_ concentration and vascular calcification in dialysis patients. This was the first report revealed relationship between S_Ox_ and vascular calcification in dialysis patients as far as we know. Previous studies^20,21^ have revealed that excess S_Ox_ combines with calcium to form calcium oxalate crystals that are deposited in various tissues; for example, myocardium, renal tubules, and interstitium. In uremic atherosclerosis mice, excess S_Ox_ has been shown to alter intracellular calcium to increase in endothelial cells, promote oxidative stress, severely inhibit proliferation and migration of human endothelial cells, and induce endothelial injury^23^. Excess S_Ox_ is also correlated with aortic calcification containing a major oxalate component in the aortic wall in uremic mice^24^. Although the mechanism underlying how excess oxalate promotes vascular calcification is unknown, these previous findings support our present results.

In this retrospective study, we found that S_Ox_ concentration was associated with CVD events in dialysis patients. Recently, Pfau et al*.*^22^ reported that S_Ox_ concentration was associated with CVD mortality in 1108 dialysis patients over a 2.5-year observation period. Our findings are similar to those of Pfau et al*.*; however, in our study we adjusted for factors for mineral and bone disorder factors as covariates, whereas Pfau et al*.* did not. We think it is important to adjust for mineral and bone disorder factors because they are currently considered some of the most powerful factors that promote vascular calcification in ESRD patients^1^. Our present findings are the first to reveal an association between S_Ox_ and CAC or vascular calcification. These findings are supported by our analysis selecting factors that are already known risk factors for vascular calcification or CVD events, for example, mineral and bone disorder factors or LDL cholesterol. In addition, our study also revealed a relationship between S_Ox_ and CVD events, which is consistent with the findings from other study^22^.

Although we found that higher S_Ox_ concentration was associated with vascular calcification and CVD events, it is important to note that many factors are involved in these outcomes. In other words, traditional factors, for example those related to mineral and bone disorder^8^ or elevated LDL-cholesterol^25^, are also important to prevent vascular calcification or CVD events in dialysis patients. Some gut microbes, for example, *Oxalobacter formigenes*, *Lactobacillus*, and *Bifidobacterium* produce specific enzymes that help in the degradation of oxalate salts; humans produce no enzymes for oxalate biotransformation^26^. The use of probiotics might be a logical treatment for lowering S_Ox_. Indeed, *Oxalobacter formigenes* intervention lowered S_Ox_ concentrations in uremic atherosclerosis mice, however, it did not significantly improve vascular calcification^24^. This result indicated that vascular calcification in ESRD patients is the result of many interrelated factors, not only oxalate, multiple target therapies will likely be needed for optimal prevention for vascular calcification or CVD events.

There are several limitations to the present study. The first is that we measured S_Ox_ concentrations retrospectively by using serum that had been in storage for around 10 years. As ascorbate converts nonenzymatically to oxalate at pH > 4, it is recommended that samples are immediately cooled and acidified to lower pH to halt this biochemical process^27,28^. However, a recent study has revealed that immediate freezing without acidic conditions and maintaining the samples at − 80 °C has been shown provide accurate and stable S_Ox_ assessments for up to 21 months^28^. The serum samples used in the present study were frozen immediately upon collected and stored at − 80 °C until analysis with strict thermal management. The second limitation is that this was a retrospective study in which confounding factors were not fully considered. For example, smoking and C-reactive protein are also associated with CVD events in ESRD patients^29^, but we were unable to add them as covariates because many of the patient records were missing these values. Moreover, the study’s sample size was relatively small. The final limitation is that this study might contain sampling bias. The study participants voluntarily underwent the initial atherosclerosis checkup, meaning they may comprise a group of particularly health-conscious individuals. Although, the study population was similar to the whole Japanese dialysis population reported in 2012^30^ in terms of age, gender, dialysis duration, and diabetes mellitus, the estimated 5-year mortality of the present study population was 82.3% (95%CI, 72.9–92.9%), whereas that of the whole Japanese dialysis population was 60.0%^31^. It should be noted that the prognosis of even health-conscious ESRD patients is poorer than that of the general population, meaning that strategies to lower S_Ox_ concentrations may bring about desirable outcomes even in well-managed ESRD patients.

High S_Ox_ concentration was associated with Agatston CAC score ≥ 1000, major artery calcification volume, and new-onset CVD events in Japanese ESRD patients. A novel strategy for S_Ox_ control may bring a better prognosis to ESRD patients.

## Methods

### Study design and population

**Figure 3:**
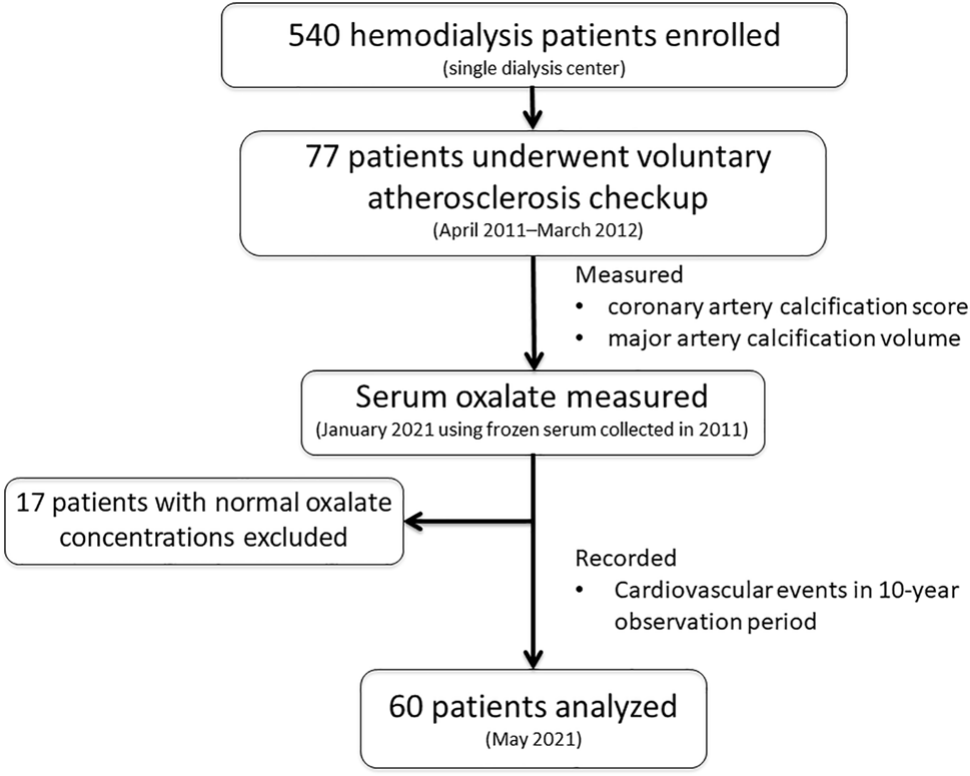
Study protocol.

A total of 540 ESRD patients undergoing maintenance hemodialysis at a single hemodialysis center (Heisei Hidaka Clinic, Gunma, Japan) were enrolled in the study (Fig. 3). All subjects were receiving hemodialysis administered in 3.5–4.5 h sessions three times weekly using a polysulfone hollow-fiber dialyzer (APS-SA, Asahi Kasei Medical, Tokyo, Japan or NV-U, Toray Industries, Tokyo, Japan) and a membrane area of 0.8–2.5 m^2^. Blood and dialysate flows were 180–250 mL/min and 500 mL/min, respectively, with a constant ultrafiltration rate. The dialysate bath comprised 140 mmol/L sodium, 2.0 mmol/L potassium, 2.5 mmol/L calcium, 1.0 mmol/L magnesium, 8.0 mmol/L acetate, 25.0 mmol/L bicarbonate, and 150 mg/dL glucose (Kindaly 3D; Fuso, Osaka, Japan). From April 2011 to March 2012, 77 subjects voluntarily received an atherosclerosis checkup in which the patients were examined by multidetector spiral computed tomography (MDCT), and Agatston CAC score^4^ and major artery calcification volume were determined by a skilled radiologist; further details are provided in the section titled “MDCT and Measurement of CAC and Major Artery Calcification Volume”. We did not calculate the sample size. We included all the participants who voluntarily received an atherosclerosis checkup from April 2011 to March 2012 with consent (N = 77) to this study. Patients without consent were excluded. All of the study participants provided written informed consent at the time of the atherosclerosis checkup in 2011, and we afforded them the opportunity to opt-out from this secondary analysis in 2021. The data were collected by using the hospital chart from January 2021 to May 2021. The study complies with the Declaration of Helsinki, and the study was approved by the Ethics Committee on Human Research at Heisei Hidaka Clinic (Gunma, Japan; No. 46) and the Ethics Committee on Clinical Research at Teikyo University (Tokyo, Japan; No. 22-058). Confirms that all experiments were performed in accordance with relevant named guidelines and regulations.

### Cross-sectional analysis of the association between S_Ox_ and vascular calcification

In January 2021, S_Ox_ concentration was measured retrospectively by a laboratorian not directly involved in the planning of the study by using frozen serum collected around the atherosclerosis checkup date and stored at − 80 °C. Because our study targeted subjects with higher S_Ox_ concentrations, we excluded patients with S_Ox_ concentrations in the normal range (< 181 µmol/L) at the time of analysis. Detailed methods of S_Ox_ measurement were provided in the “Sample handling and measurement of S_Ox_” section.

To examine the association between S_Ox_ and vascular calcification, we conducted a cross-sectional analysis using data collected at the atherosclerosis checkup (age, gender, hemodialysis duration, dialysis prescriptions, past medical history, medications, BMI, ankle-brachial index, CAC score, and major artery calcification volume). Laboratory data were also collected from medical records at the start of the dialysis session with the longest interdialytic interval around the atherosclerosis checkup date. We divided subjects into two groups: those with CAC score < 1000 and those with CAC score ≥ 1000 to define “very high” CAC^5–7^ group. The predictor was S_Ox_, and outcome was Agatston CAC score ≥ 1000. A logistic regression analysis was performed for the association between S_Ox_ and CAC score ≥ 1000. We further analyzed the relationship between S_Ox_ and major artery calcification volume in addition to the relationship between S_Ox_ and CAC. Potential confounders^8,25,29,32–36^ were age, gender, hemodialysis duration, history of CVD, diabetes mellitus, BMI, ankle-brachial index, albumin-adjusted calcium, serum phosphate, intact parathyroid hormone, alkaline phosphatase, serum magnesium, triglyceride, LDL cholesterol, HDL cholesterol, uric acid, beta-2 microglobulin, blood urea nitrogen, hemoglobin, serum albumin, vitamin D medication, calcium carbonate intake, and statin intake.

### Retrospective cohort analysis of the relationship between S_Ox_ and new-onset CVD events

To examine the relationship between S_Ox_ and new-onset CVD events, we conducted a retrospective cohort analysis. New-onset CVD events during the 10-year period from serum sample collection date to observation end date of May 15th, 2021 were recorded by medical staff blinded to the S_Ox_ results to avoid information bias. “New-onset CVD event” was defined as any admission due to non-fatal myocardial infarction, coronary artery disease, or heart failure during the observation period. We divided subjects into two groups by S_Ox_ median and compared new-onset CVD events between two groups. The explanatory variable was S_Ox_ lower or grater than median, and outcome was new-onset CVD event. Potential confounders were the same as listed on the “Cross-sectional Analysis of the Association Between S_Ox_ and Vascular Calcification” section**.**

### Sample handling and measurement of S_Ox_

Blood samples were collected at the start of the dialysis session with the longest interdialytic interval around the atherosclerosis checkup date. The collected samples were mixed immediately with 5 mg edetic acid, centrifuged at 3000 rpm for 5 min to separate the serum, and stored immediately at − 80 °C with strict thermal management until analysis. S_Ox_ concentration was measured in January 2021 by using an Oxalate Assay Kit (Colorimetric) (ab196990; Abcam, Cambridge, MA, USA). With this assay kit, the normal range of human S_Ox_ is < 181 µmol/L. All serum samples were analyzed in duplicate.

### MDCT and measurement of CAC and major artery calcification volume

Multi-slice computed tomography (CT) scans were performed with an Aquilion TSX-101A (Toshiba, Tokyo, Japan). Slices of 1.25-mm thickness were acquired eight at a time under the following conditions: 120–140 kVp, 85–150 mA, 500 ms exposure, and 0.5 s gantry rotation time. The entire heart was covered in a single breath-hold (20–30 s). The CT images were transferred to a Ziosoft M900 QUADRA workstation (AMIN, Inc, Tokyo, Japan) and CAC score according to the algorithm suggested by Agatston et al.^4^ (area × cofactor; 1: 130–199H; 2: 200–299H; 3: 300–399H; 4: > 400H), along with the volume (area × slice increment), mass (area × slice increment × mean CT density/250), and density (mass/volume) of the CAC were determined by a single radiologist. Using the electrocardiogram tracing, the workstation software automatically selected a reduced set of diastolic images from each cardiac cycle. All pixels with density > 130H were automatically highlighted on the images. The radiologist assigned one of four locations to each calcified plaque: left main, left anterior descending, circumflex, or right coronary artery. The score for each plaque equaled the plaque area × weighting factor × increment/slice width. The score for the entire specimen equaled the sum of the scores for each plaque. The mean intra-reader variability for CAC was 1.8%.

Quantification of major artery calcification volume was performed by three-dimensional calcified lesion reconstitution from multiple slices of the aorta from the top of the arch to the abdominal artery just before the bifurcation of the iliac artery along the longitudinal axis by using the Ziosoft M900 QUADRA workstation.

### Statistical analysis

Continuous variables are reported as mean ± standard deviation for normally distributed data, or median (inter-quartile range) for non-normally distributed data. Discrete variables are expressed as numeral (percentage). Differences between subjects with CAC score ≥ 1000 and those with CAC score < 1000 were tested for statistical significance by using Welch’s *t*-test for normally distributed data, the Wilcoxon rank sum test for non-normally distributed data, and Fisher’s exact test for discrete variables. A two-tailed *P* value of < 0.05 was considered to indicate statistical significance. In univariate analyses, we did available-case analyses, whereas in multivariable analyses, we did complete-case analyses. No imputation methods for sensitivity analysis were used in our study.

In the analysis of an association between S_Ox_ concentration and CAC score, we divided subjects into two groups: those with CAC score < 1000 and those with CAC score ≥ 1000. This cutoff value was selected from previous studies indicating that CAC score ≥ 1000 is associated with cumulative incidence of cardiovascular events^5–7^. A logistic regression analysis was performed for the association between S_Ox_ and CAC score ≥ 1000, and a linear regression analysis was performed for the association between S_Ox_ and major artery calcification volume. Moreover, we performed risk prediction modeling to predict CAC score ≥ 1000 for validation. We present a ROC curve for predicting CAC score ≥ 1000 by using variables selected from multivariable logistic regression with variable selection.

In the analysis of an association between S_Ox_ and new-onset CVD events, we divided subjects into two groups by S_Ox_ median and performed a Kaplan–Meier analysis and log-rank test to compare the survival functions of the two groups and to determine whether S_Ox_ is associated with CVD events. We repeated the analysis, restricting it to patients with CAC score < 1000 and without history of CVD. We also conducted a Cox proportional hazard analysis using the S_Ox_ cut-off value to determine which factors were associated with CVD events.

In each analysis, the explanatory variable was S_Ox_ and the covariates were factors that are reported to be associated with vascular calcification^8,25,32,33^ or CVD^29,34–36^; specifically, age, gender, hemodialysis duration, history of CVD, diabetes mellitus, BMI, ankle-brachial index, albumin-adjusted calcium, serum phosphate, intact parathyroid hormone, alkaline phosphatase, serum magnesium, triglyceride, LDL cholesterol, HDL cholesterol, uric acid, beta-2 microglobulin, blood urea nitrogen, hemoglobin, and serum albumin, vitamin D medication, calcium carbonate intake, and statin intake were used as covariates. A univariable analysis was performed first, and factors with a two-tailed *P* value < 0.2 were used for a multivariable analysis. For variable selection, we used Akaike’s information criteria with stepwise backward elimination. All statistical analyses were performed using R software, version 4.1.3.

## Data Availability

The datasets generated during and/or analyzed during the current study are available from the corresponding author on reasonable request.
